# Silent but Significant: A Case of Indwelling Chemoport-Associated Eustachian Valve Endocarditis

**DOI:** 10.7759/cureus.92736

**Published:** 2025-09-19

**Authors:** Grace V Ralston, Saroj K Jha, Matthew Kwon

**Affiliations:** 1 Internal Medicine, Edward Via College of Osteopathic Medicine, Spartanburg, USA; 2 Internal Medicine, Cleveland Clinic Akron General, Akron, USA

**Keywords:** endovascular endocarditis, eustachian valve endocarditis, long-term indwelling catheters, mrsa bacteremia, right-sided infective endocarditis

## Abstract

Eustachian valve endocarditis (EVE) is a rare form of endovascular endocarditis, often associated with the use of indwelling venous catheters. We present a case of a 75-year-old immunocompromised female with multiple comorbidities, including a history of methicillin-resistant *Staphylococcus aureus* (MRSA) septic joint infection, who was admitted for acute hypoxic respiratory failure secondary to a chronic obstructive pulmonary disease exacerbation. Blood cultures revealed MRSA bacteremia resistant to clindamycin, prompting initiation of intravenous vancomycin. Transthoracic echocardiography (TTE) suggested a possible mobile density in the left ventricle, though limited by body habitus. Transesophageal echocardiography (TEE) confirmed a 0.8 x 0.2 cm mobile echodensity on the eustachian valve, consistent with EVE. The source of infection was suspected to be the patient’s indwelling chemoport, whose catheter tip terminated adjacent to the eustachian valve. The port was surgically removed, and cultures from the catheter tip were negative. The patient was treated with eight weeks of intravenous antibiotics, initially with daptomycin, which was discontinued due to drug-induced fever, necessitating re-initiation of vancomycin. This case underscores the importance of considering non-valvular endovascular sites, such as the eustachian valve, in patients with persistent bacteremia, particularly in the presence of intravascular devices. While EVE often presents asymptomatically and is diagnosed incidentally, TEE remains the diagnostic modality of choice due to its superior visualization of vegetations, especially in patients with suboptimal TTE imaging windows. Source control through removal of infected hardware and tailored antimicrobial therapy based on pathogen susceptibility and patient tolerability remains the cornerstone of management. This case highlights the importance of heightened clinical suspicion and a multidisciplinary approach to achieve successful outcomes in rare presentations of endocarditis.

## Introduction

Infective endocarditis is an infection involving the endocardium, most commonly including one or more heart valves, for which it is referred to as infective endocarditis. Valvular endocarditis is the most common type of endocarditis, with left-sided involvement more common than right-sided valvular disease. Left-sided endocarditis is mostly associated with rheumatic heart disease, degenerative valve disease, prosthetic heart valves, and patients with poor dentition. Right-sided endocarditis is more commonly associated with intravenous drug use, implantable cardiac devices, and indwelling central lines.

Endovascular endocarditis, a type of non-valvular endocarditis, involves the cardiovascular endothelial surfaces, such as the mural endocardium, and is commonly due to intracardiac devices and indwelling venous catheters [1]. Eustachian valve endocarditis (EVE) is a rare type of right-sided endovascular endocarditis. The eustachian valve is an uncommon embryologic remnant of the sinus venosus, which is located at the junction of the inferior vena cava and right atrium that plays a role in directing oxygenated blood in fetal circulation from the inferior vena cava to the left atrium through the foramen ovale [1-3]. It was first described in 1986 by Edwards et al. [4], with only 58 publications contributing to medical literature as of recently. Due to its nonspecific presentation and rare nature, diagnosis often requires a high clinical suspicion when it is not found incidentally. This report documents the diagnostic workup, source control, and management of a 75-year-old female with asymptomatic EVE.

## Case presentation

A 75-year-old Caucasian female was admitted to the hospitalist service for treatment of acute hypoxic respiratory failure secondary to a chronic obstructive pulmonary disease (COPD) exacerbation, with additional concern for possible left knee cellulitis. Her past medical history included class III morbid obesity, pulmonary hypertension, multiple myeloma, hypogammaglobulinemia, hypertension, hyperlipidemia, obstructive sleep apnea, heart failure with preserved ejection fraction, paroxysmal atrial fibrillation and flutter, and renal cell carcinoma in remission. She had a methicillin-resistant *Staphylococcus aureus* (MRSA)-positive prosthetic septic joint infection involving the same left knee five months prior, which was managed outpatient with lifelong suppressive clindamycin therapy.

Upon admission, physical examination of the lower left extremity revealed no signs concerning for cellulitis or septic joint. Given the patient’s history of prior MRSA infection, blood cultures and nares swabs were obtained. Both resulted in positive results for MRSA and demonstrated resistance to clindamycin, oxacillin, gentamicin, and tetracycline. Clindamycin was discontinued, and empiric coverage with intravenous vancomycin was initiated.

**Figure 1:**
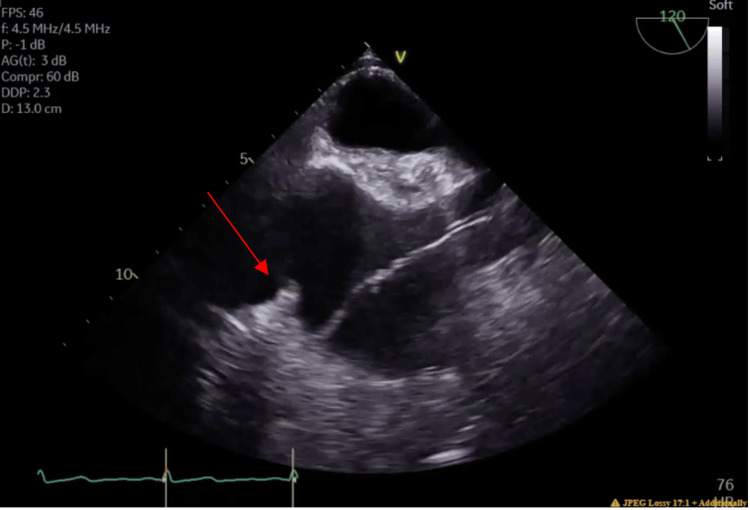
Transesophageal echocardiogram demonstrating 0.8 x 0.2 cm mobile echodensity (red arrow) attached to the eustachian valve.

A transthoracic echocardiogram (TTE) was performed in the context of MRSA bacteremia, demonstrating a left ventricular ejection fraction (LVEF) of 55% and a possible, though not definitive, mobile density associated with the lateral wall of the left ventricle. Her LVEF one month prior was 65%. The remainder of the TTE was limited by body habitus. A transesophageal echocardiogram (TEE) was performed to further evaluate the suspected mobile density, which demonstrated a 0.8 x 0.2 cm mobile echodensity attached to the eustachian valve (Figure 1). The patient had a right-sided chemo chest port placed; however, no obvious echodensities were observed attached to the venous catheter present in the right atrium, and there was no evidence of any valvular vegetations. To rule out the presence of a patent foramen ovale, an agitated saline study was performed with no evidence of intracardiac shunting. The patient was diagnosed with endovascular endocarditis involving the eustachian valve.

The source of infection was suspected to be the chronic indwelling port, as the catheter tip terminated in the right atrium adjacent to the eustachian valve. The port was surgically removed, and the catheter tip was sent for culture. While the patient remained on empiric vancomycin, both repeat blood and catheter tip cultures returned negative. Infectious disease specialists recommended long-term outpatient antibiotic therapy with daptomycin 700 mg for a total duration of eight weeks from the first set of negative blood cultures. The patient received two doses of daptomycin inpatient, unfortunately resulting in a drug-induced fever. Daptomycin was discontinued, and the patient was restarted on intravenous vancomycin to be continued outpatient for the remainder of the eight weeks of treatment.

## Discussion

The tricuspid valve is most implicated in right-sided endocarditis; however, cases such as EVE highlight the importance of considering endovascular structures beyond the valves. From a cardiology perspective, our patient was asymptomatic, presenting with no stigmata indicative of endocarditis. Most cases of EVE are incidentally found during echocardiogram workup for persistent bacteremia rather than clinical suspicion alone [3,5]. Our patient’s case of EVE was confirmed with TEE. Current diagnostic and treatment guidelines for valvular and non-valvular endocarditis recommend TEE based on its superiority compared to TTE in visualizing vegetations, especially when body habitus limits the TTE window [1,5,6].

Since EVE was discovered in 1986 [4], central venous catheters have been implicated as the source of infection in several of the reported cases [2,5-7]. The tip of the indwelling port in our patient was suspected to be the likely source of infection due to its termination point adjacent to the eustachian valve in the right atrium. There are no specific guidelines for the duration of therapy in EVE; however, most reported cases follow the guidelines for right-sided endocarditis, including six to eight weeks of intravenous antibiotics from the date of the first negative blood culture and removal of infected hardware for source control when possible [6-8]. *Staphylococcus aureus*, notably MRSA, is the most frequently encountered pathogen in right-sided endocarditis, as seen in our patient. While daptomycin has been reported as an effective alternative in right-sided endocarditis [8], especially in those with higher risks of renal injury, our patient developed a daptomycin-induced fever, resulting in re-initiation of vancomycin. Antimicrobial resistance is always a concern when treating cases of endocarditis, but it is also important to tailor therapy to a patient’s drug tolerability to improve adherence and overall outcomes.

## Conclusions

This case documents the successful diagnosis and management of asymptomatic MRSA-associated EVE in an immunocompromised patient with multiple comorbidities. It is important for cases with persistent bacteremia, particularly when intravascular devices are present, to maintain a high index of suspicion for non-valvular sites of infection. TEE remains the preferred diagnostic modality for endocarditis, especially in those with body habitus that limits TTE windows, and source control with appropriate antimicrobial intervention remains the mainstay of treatment.

## References

[1] Skaff P, Kim C, Benjamin MM (2020). Eustachian valve endocarditis: its presentation and clinical characteristics. J Cardiol Cases.

[2] Reyes D, Musleh G, Elkattawy S (2024). Eustachian valve endocarditis: a case report and literature review. J Community Hosp Intern Med Perspect.

[3] Cresti A, Baratta P, De Sensi F, D'Aiello I, Costoli A, Limbruno U (2017). Frequency and clinical significance of right atrial embryonic remnants involvement in infective endocarditis. J Heart Valve Dis.

[4] Edwards AD, Vickers MA, Morgan CJ (1986). Infective endocarditis affecting the eustachian valve. Br Heart J.

[5] Hasan S, Blanco A, Faluk M, Nasser H (2020). Eustachian valve endocarditis: a case report on an under diagnosed entity. J Community Hosp Intern Med Perspect.

[6] Hernandez SD, Jabaay MJ, Marotta DA, Tosto ST, Velayati A (2021). Thoracic osteomyelitis and eustachian valve endocarditis: a case report and literature review. Cureus.

[7] Mahamid M, Mashiah J, Rozner E, Jabaren M, Turgeman Y, Koren O (2020). Right-sided endocarditis involving eustachian valve following the use of a central venous line. Am J Case Rep.

[8] Kumar D, Boyer J, Fnu W, Boamah H (2021). Case of eustachian valve endocarditis and the importance of synergistic antibiotic therapy. BMJ Case Rep.

